# 

**Published:** 1908-09-05

**Authors:** 


**THE HOSPITAL** September 5, 1908.

*Name* ..........................................................................

*Address* ..........................................................................

..........................................................................

This Coupon must accompany manuscript or contributions intended for The Hospital.

### JOHN WRIGHT & SONS Ltd., Publishers, BRISTOL.

New Series. Enlarged. Copiously Illustrated, with many Full-page Plates. 26th Year. 8s. 6d. net. Stereoscope, 2s. or 2s. 6d. extra.

### THE MEDICAL ANNUAL, 1908.

**A Year-Book of Treatment and Practitioners' Index.**

A Complete Work of Reference, alphabetically arranged, combining an Annual Retrospect with a Medical Encyclopædia.

"Of great utility—a trustworthy work of reference."—*Brit. Med. Journal.* "Of special benefit to those in general practice—will often provide a suggestion for treatment at the moment it is needed."—*Lancet.*

Nearly ready. Crown 8vo. 7s. 6d. net.

### SYNOPSIS OF SURGERY.

By ERNEST W. HEY GROVES, M.S., F.R.C.S., Assistant Surgeon, Bristol General Hospital; Surgeon to Cossham Hospital; Senior Demonstrator of Anatomy at University College, Bristol.

The volume is an attempt to present an epitome of the salient facts in surgical practice in such a manner that they may most easily be referred to or revised. It is obvious that large works cannot be dispensed with, but students and busy practitioners may find this synopsis useful to refer to as an aid to the memory in retaining a large array of facts in an orderly manner.

Eleventh Edition. 60th Thousand. Cloth, 2s. 6d.; paper, 1s. 6d. MAY BE SAFELY RECOMMENDED.

### OUR BABY: a Book for Mothers and Nurses.

By Mrs. LANGTON HEWER (late Hospital Sister).

"We can heartily recommend it."—*British Medical Journal.* "The best we have ever seen."—*Edinburgh Medical Journal.* "Simply invaluable. Young mothers should study this volume attentively."—*Queen.*

**THE CRUSADE AGAINST CONSUMPTION.** Second Edition. Complete with Sample Chart, 1s. net.

### CONSUMPTION: Treatment at Home and Rules for Living.

By H. WARREN CROWE, M.D.

This booklet may be placed in the hands of patients who, by learning these rules, save the practitioner's time and become grounded in the first elements of their successful treatment.

Sixth Edition. Revised and Enlarged. Many Illus. 454 pp. 7s. 6d. net.

### LECTURES ON MASSAGE AND ELECTRICITY IN THE TREATMENT OF DISEASE.

By T. STRETCH DOWSE, M.D.

Third Edition. Strongly bound to resist damp. 3s. 6d. net.

### DISPENSING MADE EASY. With numerous

Formulæ and Practical Hints to secure Simplicity, Rapidity, and Economy. By WM. G. SUTHERLAND, M.B.Aberd.

Waistcoat Pocket Size, cloth limp, 1s. each.

### "GOLDEN RULES" SERIES: IN MEDICINE AND SURGERY.

1.—GOLDEN RULES OF SURGICAL PRACTICE. By E. HURRY FENWICK, F.R.C.S. 9th Edition, Revised and Enlarged.
2.—GOLDEN RULES OF GYNÆCOLOGY. By S. JERVOIS AARONS, M.D. 5th Edition.
3.—GOLDEN RULES OF OBSTETRIC PRACTICE. By W. E. FOTHERGILL, M.A., B.Sc., M.D. 5th Edition.
4.—GOLDEN RULES OF MEDICAL PRACTICE. By LEWIS SMITH, M.D., M.R.C.S. 7th Edition. Enlarged and entirely Rewritten.
5.—GOLDEN RULES OF PSYCHIATRY. By JAMES SHAW, M.D. 4th Edition.
6.—GOLDEN RULES OF PHYSIOLOGY. By I. WALKER HALL, M.B., Ch.B.Vict., and J. ACKWORTH MENZIES, M.D., C.M.Ed. 3rd Edition.
7.—GOLDEN RULES OF OPHTHALMIC PRACTICE. By GUSTAVUS HARTRIDGE, F.R.C.S. 4th Edition. Revised.
8.—GOLDEN RULES OF SKIN PRACTICE. By DAVID WALSH, M.D.Edin. 3rd Edition.
9.—GOLDEN RULES OF AURAL AND NASAL PRACTICE. By PHILIP R. W. DE SANTI, F.R.C.S. 3rd Edition. Revised.
10.—GOLDEN RULES OF HYGIENE. By F. J. WALDO, M.A., M.D.Cantab., D.P.H. 2nd Edition.
11.—GOLDEN RULES OF DISEASES OF CHILDREN. By GEO. CARPENTER, M.D.Lond., M.R.C.P. 3rd Edition. Enlarged Double Number, 2s.
12.—GOLDEN RULES OF REFRACTION. By ERNEST E. MADDOX, M.D., F.R.C.S.Edin. 2nd Edition.
13.—GOLDEN RULES OF DENTAL SURGERY. By CHAS. W. GLASSINGTON, M.R.C.S., L.D.S.Edin. 2nd Edition.
14.—GOLDEN RULES OF ANÆSTHESIA. By R. J. PROBYN-WILLIAMS, M.D. 2nd Edition.
15.—GOLDEN RULES OF SICK NURSING. By W. B. DRUMMOND, M.B., C.M., F.R.C.P.Edin. 2nd Edition.
16.—GOLDEN RULES OF MEDICAL EVIDENCE. By STANLEY B. ATKINSON, M.A., M.B., B.Sc., of the Inner Temple.
17.—GOLDEN RULES OF VENEREAL DISEASE. By C. F. MARSHALL, M.D., F.R.C.S.

"Aphorisms and maxims of the greatest possible use."—*Lancet.*
"Give useful hints in many emergencies."—*Hospital.*
"Most admirable examples of what can be done in the way of condensation."—*Clinical Journal.*

**Bristol: JOHN WRIGHT & SONS, Ltd. London: SIMPKIN & CO., Ltd.**
**Full Catalogue of Medical Publications on application.**

New Work. Just issued in Two Vols., 1,900 pages, Crown 8vo. 32s.

### PRACTICE AND THEORY OF MEDICINE.

Containing a complete and independent Article on every Medical Disease including Skin Affections; arranged in Dictionary Form with copious Index for Rapid Reference, for Practitioners and Students.

By **Sir W. WHITLA, M.D., LL.D.,** Senior Physician to and Lecturer on Clinical Medicine, Royal Victoria Hospital; Professor of Materia Medica and Therapeutics, Queen's College, Belfast, &c.

Publisher will supply the above Work C.P., on receipt of 25s. 6d.

By the same Author. Eighth Edition, Crown 8vo. **10s. 6d.**

### PHARMACY, MATERIA MEDICA & THERAPEUTICS.

It contains an account **of every drug in the B.P.,** as well as a section upon all the newest drugs, with an Introduction to the Art of Dispensing. Fourth Edition, 1,055 pages, 8vo. **16s.**

### DICTIONARY OF TREATMENT.

Containing an Article upon the Treatment of every Disease, Medical, Surgical, Gynæcological, or Ophthalmological. Publisher will supply both volumes C.P., on receipt of 21s.

London: HENRY RENSHAW, 356 Strand.

### NERVE DISEASES FOR STUDENTS.

By L. A. CLUTTERBUCK, M.D., B.S. (Durham), M.R.C.P. (London), L.R.C.P. & S. (Edin.), L.F.P. & S. (Glasg.), L.R.C.P. (Ireland); Hon. Physician, St. Marylebone General Dispensary; Clinical Assistant, West End Hospital for Nervous Diseases, London, &c.

**Price 3s. net, by post 3s. 3d.**

OF ALL BOOKSELLERS, OR OF THE PUBLISHERS—
THE SCIENTIFIC PRESS, LTD.,
28/29 SOUTHAMPTON STREET, STRAND, LONDON, W.C.

A LIST of the NEW PUBLICATIONS of the SCIENTIFIC PRESS, LIMITED, will be sent post free to any member of the Medical Profession on receipt of post card.

28 & 29 SOUTHAMPTON STREET, STRAND, LONDON, W.C.

